# Comparison of the prognostic value of SYNTAX score and clinical SYNTAX score on outcomes of Chinese patients underwent percutaneous coronary intervention

**DOI:** 10.1186/s12872-021-02144-w

**Published:** 2021-07-07

**Authors:** Xiao-Qin Li, Chun Yin, Xiao-Li Li, Wen-Li Wu, Kun Cui

**Affiliations:** Department of Cardiology, Chongqing General Hospital, University of Chinese Academy of Sciences, No. 108, Xingguang Road, Liangjiang New District, Chongqing, China

**Keywords:** SYNTAX score, Clinical SYNTAX score, Percutaneous coronary intervention, Clinical outcomes

## Abstract

**Background:**

Previous studies have validated the capability of SYNTAX score (SS) and clinical SYNTAX score (CSS) in the prediction of clinical outcomes in patients who have undergone PCI; however, studies on comparison of these two scoring systems in Chinese population have been sparse.

**Methods:**

To study the ability of SS and CSS in prediction of clinical outcomes of Chinese patients underwent percutaneous coronary intervention (PCI). We retrospectively calculated SS and CSS for 547 Chinese patients from a single center who underwent PCI. Patients were stratified into tertiles according to their SS and CSS. We compared the 2-year clinical outcomes in these patients stratified separately by SS and CSS tertiles.

**Results:**

The incidence of major adverse cardiac and cerebro-vascular events (MACCE) was the highest in patients with SS_HIGH_ (13.5%), comparing to 6.8% in SS_MED_ and 0% in SS_LOW_ (*p* < 0.0001). The Cox multivariable analysis showed that the SS and CSS were both strong independent predictors for MACCE [1.100 (1.069–1.133), 1.017 (1.010–1.025), both *p* < 0.0001]. The receiver operating characteristic (ROC) curves showed the areas-under-the-curves for all-cause death by CSS was slightly larger comparing to SS but not significantly (AUC SS, 0.64; AUC CSS, 0.71; *p* = 0.23).

**Conclusion:**

We concluded that both the SS and CSS were capable of risk stratification of clinical outcomes in all-comers population as well as in low and moderate risk Chinese patients undergoing PCI with CSS showing slightly better advantage.

## Background

Percutaneous coronary intervention (PCI) is the major strategy in treating coronary artery disease (CAD) which is a leading cause of human death. The Synergy between PCI with Taxus and Cardiac Surgery score (SYNTAX score, or SS) is an angiographic scoring system which is based on the complexity of coronary lesions [1, 2]. In clinical practice, SS is being used to help with the decision-making between PCI and coronary artery bypass graft (CABG) surgery and to predict the prognostic outcomes. In the SYNTAX trial [3], SS has been proven to be effective in predicting the clinical outcomes in patients with three-vessel and/or left main coronary artery disease after PCI [4, 5]. The ability of SS to risk stratify patients has been evaluated in numerous studies, including those with an all-comers design, as well as those only enrolling patients with 3-vessel and/or left main disease [6–8].

However, SS does not take into account of other clinical factors, such as those included in the ACEF (age [9], creatinine [10], and ejection fraction [11]) score, which could significantly impact the prediction of prognostic outcomes [12–14]. To overcome this limitation, clinical SYNTAX score (CSS) was proposed, which incorporates clinical variables in addition to angiographic variables. CSS had been shown to improve the predictive ability for adverse clinical outcomes in patient underwent PCI [15, 16]. However, these risk scoring models were more frequently used in high risk (3-vessel and/or left main coronary artery disease) patients treated by PCI, few studies included a study population composed of a majority of low and moderate risk patients. Moreover, CSS is sparsely tested on Chinese population. China has a population of 1.4 billion. This is almost one fifth of the world’s total population. Importantly, among 1.4 billion Chinese people, the vast majority (1.3 billion) belong to the Han people, which is the world's largest ethnicity. This situation is very different from other countries, like the US, where multiple ethnicities make up considerable percentages of the total population. Due to this huge population base and the domination of Han people, most population-based studies need to be tested in Chinese-dominated population to prove their applicability to Chinese people.

Thus, the current study was designed to address the following aims: (1) to validate and compare the performance of the SS with the CSS in all comer Chinese patients who have successfully undergone PCI; (2) to evaluate whether the CSS risk model has improved performance in prediction of adverse outcomes in patients after incorporating clinical information, especially in low and moderate risk patients after PCI.

## Methods

### Study design and patient population

In the current study, a total of 547 patients with chronic stable CAD or acute coronary syndromes who successfully received PCI procedure from June 2014 to November 2017 in the Second Affiliated Hospital of Chongqing Medical University (Chongqing, China) was enrolled. All patients had at least one lesion with diameter stenosis (DS) greater than 50% in a blood vessel suitable for stent implantation (reference diameter between 2.25 and 4.00 mm). No restrictions for the number of lesions, number of treated vessels or stents implanted was set in current study. The primary criteria for exclusion were: pregnancy; allergy to contrast media, previous history of coronary artery bypass grafting (CABG), any planned surgery within 6 months of PCI (unless the patients will maintain dual antiplatelet therapy throughout the peri-operative period); or the participation in another trial before reaching the primary endpoint.

### Procedures

All patients received anticoagulation treatment as follows: clopidogrel 75 mg/day together with aspirin 100 mg/day, more than 24 h in advance before PCI procedure. During the primary PCI, unfractionated heparin (70–100 U/kg) was used for all patients. For patients with acute coronary syndromes requiring emergency PCI, Clopidogrel 600 mg loading dose and glycoprotein IIb/IIIa inhibitors were used. The selection of drug-eluting stents and adjunctive devices was at the operators’ own discretion. To deploy stents, high pressure balloon dilatation method was used to achieve optimal stent apposition. PCI was deemed angiographically successful if residual stenosis was less than 30% and the restoration of coronary TIMI (thrombolysis in myocardial infarction) 3 flow at the end stage of the procedure. Five out of 547 patients had coronary dissection, 28 out of 547 patients had side-branch occlusion, coronary perforation cases were rare and considered unsuccessful PCI, thus were not included in the study. All discharged patients were prescribed with clopidogrel 75 mg/day for at least 12 months together with lifelong aspirin at a dose of 100 mg/day. Patients were requested to return for a routine in-hospital follow-up at 1 month, 6 months and 12 months after discharge and additional telephonic follow-ups were performed for up to 2 years. Medical records were reviewed in addition to telephone follow-up of patients to confirm the occurrence of endpoints.

### Calculation of SS and CSS

Two cardiologists adjudicated the angiographic features of each patient with very good agreement (kappa index = 0.81). In case discrepancies occur, a third cardiologist was invited to reach consensus. The SS for each patient was calculated by scoring all coronary lesions with a DS ≥ 50% in vessels ≥ 2.25 mm as has been instructed on the SYNTAX score website (www.syntaxscore.com). The demographic characteristics of each participating patient, together with their history of heart diseases, and related risk factors were collected and archived upon admission. The Age, Creatinine, and Ejection Fraction (ACEF) score was calculated by patients' left ventricular ejection fraction, age, and creatinine clearance [16]. The CSS was calculated by multiplying the modified ACEF score with SS (CSS = [SS] × [modified ACEF score]) according to previous publications [16].

### Endpoint and definitions

The primary endpoint of this study included cardiac death, stroke, Myocardial infarction (MI) and any repeat revascularization (either PCI or CABG). The detailed definitions of each endpoints in current study were provided as below. All deaths were considered cardiogenic death unless a definite non-cardiogenic cause was recorded. Stroke was diagnosed by neurologists and was defined as a focal neurological deficit of vascular origin lasting for longer than 24 h. MI was defined according to definitions from Academic Research Consortium as the presence of new Q-waves of at least 0.4 s duration in ≥ 2 contiguous leads and elevation of cardiac enzyme. In those patients without pathologic Q-waves, MI was defined as the elevation of the creatine kinase level to > 2x of the upper limit of normal range, together with elevated level of creatine kinase MB or troponin I. Target-vessel revascularization or TLR was defined as an interventive procedure of either PCI or CABG for treatment of a stenosis within previous PCI treated vessel. Ischemia driven any repeat revascularization was defined by lumen stenosis ≥ 50% angiographically in the presence of ischemic symptoms, or stenosis ≥ 70% regardless of ischemic symptoms or signs. Stent thrombosis was defined as probable or definite, according to the recommendation of Academic Research Consortium.

### Statistical analysis

Statistical analysis was performed using the Windows version of SPSS (ver. 19.0 SPSS, Inc, Chicago, IL, USA). All patients with calculated SS and CSS scores were included in the analysis. All continuous variables were in the form of mean and standard deviation (SD). All categorical variables were shown as counts and percentages. The Kolmogorov–Smirnov test was used for normality assessment of the SS and CSS distribution. All variables were stratified according to SS/CSS tertiles. The correlation between SS and CSS was assessed by Spearman’s test. Comparisons for continuous variables with a normal distribution were performed by one-way analysis of variance (ANOVA) and Chi-square test was used for all categorical variables. Time to event trends were demonstrated as Kaplan–Meier curve, and Log-rank test was used to assess the differences in survival among subgroups of patients.

The prognostic value of the SS and CSS was assessed by plotting the receiver-operator characteristic curves (ROCs), in which 0.50 indicates no discrimination and 1.0 indicates perfect discrimination. Areas-under-the-curves (AUCs) for SS and CSS were compared with the DeLong method using Windows version of MedCalc (ver. 11.6.0.0 MedCalc Software). Finally, to evaluate whether CSS is superior in the risk stratification comparing to SS, a net reclassification improvement analysis was performed.

To rule out potential confounders, univariable and multivariable Cox proportional hazard regression models was used to assess the relationship between SS/CSS and the incidence of primary endpoint. The variables were chosen according to their clinical significance. The following variables including sex, diabetes mellitus (DM), estimated glomerular filtration rate (eGFR), smoking, acute coronary syndrome (ACS), number of stents implanted, were tested on a per patient basis to determine suitability for inclusion in the multivariate model by univariate analysis.

All statistical tests were 2-tailed, and a *p* value of less than 0.05 were considered as statistically significant.

## Results

### Baseline clinical characteristics

Baseline clinical and risk factors of the study population, stratified according to SS tertile were presented in Table 1.

**Table 1:** Baseline clinical characteristics and risk factors

Variable	SS_low_ ≤ 8 (177)	8 < SS_mid_ < 18 (177)	18 ≤ SS_high_ (193)	Total	*p* value
Baseline characteristics					
Male sex	108 (61.0%)	123 (69.5%)	142 (73.6%)	373 (68.2%)	0.031
Age (years)	65.11 ± 4.00	68.20 ± 8.19	66.92 ± 3.00	66.82 ± 3.50	0.045
Body mass index (kg/m^2^)	24.26 ± 3.07	23.52 ± 2.98	23.97 ± 3.12	23.67 ± 2.12	0.211
Risk factors					
Left ventricular ejection fraction	67.17 ± 10.38	69.74 ± 8.66	65.71 ± 9.68	67.71 ± 7.63	< 0.001
Creatinine clearance, ml/1.73 m^2^	64.41 ± 37.32	57.41 ± 25.14	56.53 ± 26.87	58.53 ± 16.65	0.06
Creatinine > 100 μmol/l	33 (18.6%)	44 (24.9%)	66 (34.2%)	144 (26.3)	0.003
Hypertension	125 (70.6%)	116 (65.5%)	127 (65.8%)	370 (67.6%)	0.513
Diabetes mellitus	48 (27.1%)	59 (33.3%)	52 (26.9%)	159 (29.1%)	0.315
Hypercholesterolemia	67 (37.9%)	73 (41.2%)	82 (42.5%)	222 (40.6%)	0.648
Current smoker	71 (40.1%)	60 (33.9%)	89 (46.1%)	220 (40.2%)	0.057
Stroke	24 (13.6%)	13 (7.3%)	20 (10.4%)	57 (10.4%)	0.16
Chronic pulmonary disease	17 (9.6%)	21 (11.9%)	24 (12.4%)	62 (11.3%)	0.667
Previous MI	9 (5.1%)	15 (8.5%)	8 (4.1%)	32 (5.9%)	0.181
Previous PCI	6 (3.4%)	11 (6.2%)	3 (2.6%)	20 (3.6%)	0.182
SS	4.81 ± 2.21	12.86 ± 2.66	26.07 ± 6.83	14.90 ± 8.83	< 0.0001
Indication for treatment					
Stable angina	60 (33.9%)	76 (42.9%)	69 (35.8%)	205 (37.4%)	0.177
Unstable angina	53 (29.9%)	41 (23.2%)	57 (29.5%)	151 (27.6%)	0.174
ST-segment elevation MI	27 (15.3%)	26 (14.7%)	34 (17.6%)	87 (15.9%)	0.714
Non-ST-segment elevation MI	15 (8.5%)	14 (7.9%)	7 (3.6%)	36 (6.6%)	0.118
Silent ischemia	22 (12.4%)	19 (10.7%)	27 (14.0%)	27 (12.4%)	0.638
Medication at discharge					
Aspirin	144 (81.4%)	141 (80.1%)	172 (89.1%)	457 (83.5%)	0.038
Clopidogrel	139 (78.5%)	149 (84.2%)	164 (85.0%)	452 (82.6%)	0.211
β-Blocker	88 (49.7%)	93 (52.5%)	90 (46.6%)	90 (49.5%)	0.524
Statin	139 (78.5%)	146 (82.5%)	165 (85.5%)	271 (85.5%)	0.215
ACEI/ARB	82 (46.3%)	92 (52.0%)	82 (42.5%)	256 (46.8%)	0.186

Value are n (%), mean ± SD

*MI* myocardial infarction, *PCI* percutaneous coronary intervention, *SS* SYNTAX score, *SYNTAX* Synergy Between Percutaneous Coronary Intervention with Taxus and Cardiac Surgery, *ACEI* angiotensin converting enzyme inhibitors, *ARB* angiotensin receptor blocker

**Fig. 1:**
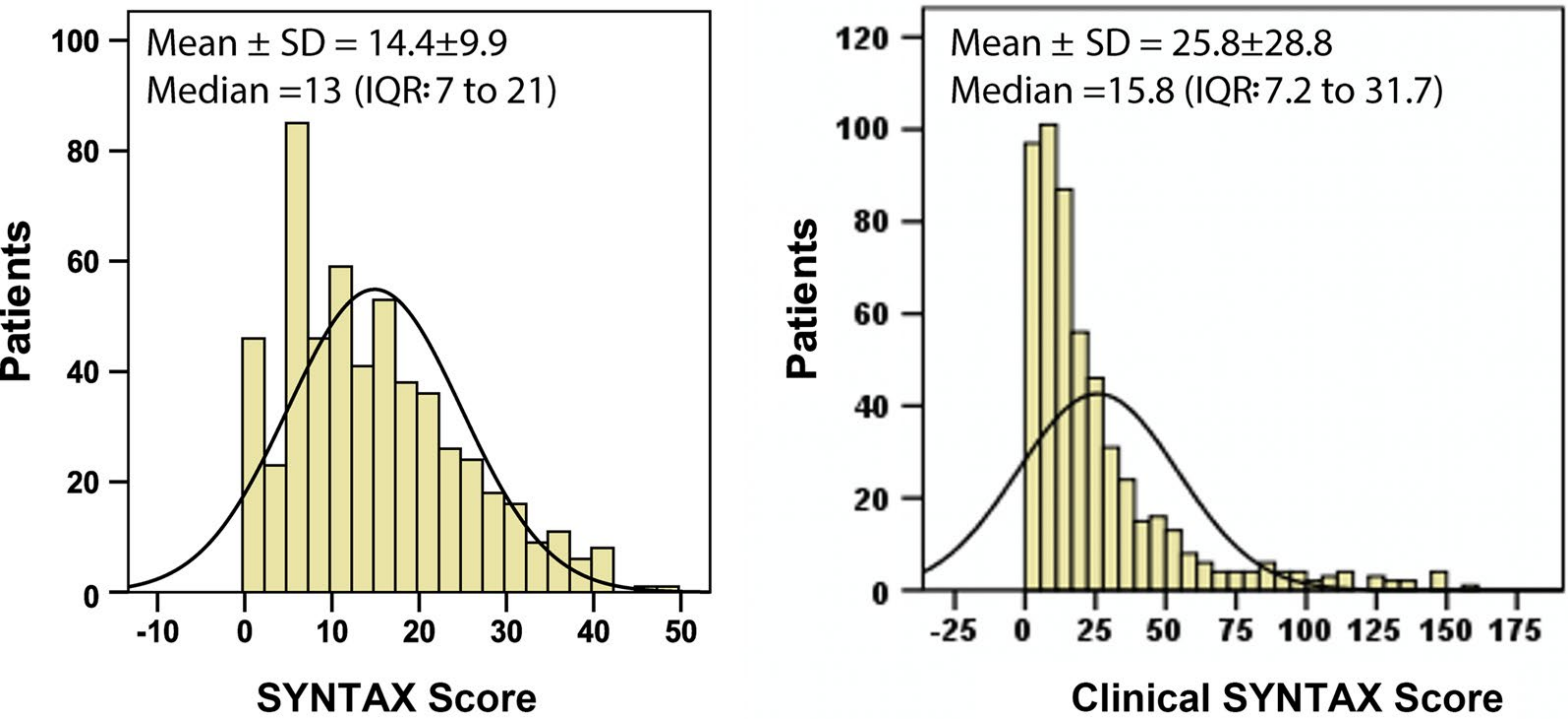
Distribution of SS and CSS among the 547 patients enrolled in the study. Histograms of the SS (left side) and CSS (right side) with a superimposed normal curve. Both score distributions are skewed to right, and not normally distributed. Mean ± SD values and median values plus inter-quartile range (IQR) are reported

The SS was available for 547 patients (1314 lesions in total, with an average of 2.4 ± 1.4 lesions for each patient) enrolled in current study. Overall, the SS ranged from 1 to 47.5, with a mean of 14.90 ± 8.83 and a median of 13.00. The CSS ranged from 0.9 to 158.2, with a mean of 25.80 ± 15.23 and a median of 15.80. Expectedly, there was a strong correlation between the two scores (r = 0.713, *p* < 0.001). The distributions of the two scores within the study population were shown in Fig. 1. Both scores were not normally distributed (Kolmogorov–Smirnov test both *p* < 0.001) but skewed to the right. In this analysis, patients were categorized according to tertile of the SS and CSS as follows: SS_LOW_ ≤ 8 (n = 177), 8 < SS_MID_ < 18 (n = 177), 18 ≤ SS_HIGH_ (n = 193); CSS_LOW_ ≤ 10 (n = 180), 10 < CSS_MID_ ≤ 25 (n = 185), 25 < CSS_HIGH_ (n = 182), respectively.

The mean age was 66.82 ± 3.50 with 68.2% male predominance. There were 159 (29.1%) patients with diabetes mellitus, 68.3% patients with hypertension. Only 32 (5.9%) patients had myocardial infarction history. Of all enrolled patients, almost one in two patients (274/547, 50.1%) presented with ACS, among which, 151 presenting unstable angina, 87 presenting ST-Elevation Myocardial Infarction (STEMI), 36 presenting Non-ST-elevation myocardial infarction (NSTEMI).

The percentage of patients with 1-vessel, 2-vessel and 3-vessel lesion was 33.3%, 33.8% and 32.9%, respectively (Table 2). Complete revascularization was achieved in 265 (48.1%) patients, the percentage of patients underwent complete revascularization was 83.1% (147), 51.4% (91), 13.0% (25) in SS_LOW_, in SS_MED_ and in SS_HIGH_ tertile, respectively (Table 2).

**Table 2:** Angiographic and procedural characteristics stratified according to SS tertile

Variable	SS_low_ ≤ 8 (177)	8 < SS_mid_ < 18 (177)	18 ≤ SS_high_ (193)	Total	*p* value
Diseased lesions					
Number of disease lesions	1.5 ± 0.7	2.1 ± 1.0	3.3 ± 1.3	2.3 ± 1.6	< 0.001
1-Vessel disease	131 (74.0%)	46 (26.0%)	5 (2.6%)	182 (33.3%)	< 0.001
2-Vessel disease	40 (22.6%)	86 (48.6%)	59 (30.6%)	185 (33.8%)	< 0.001
3-Vessel disease	6 (3.4%)	45 (25.4%)	129 (66.8%)	180 (32.9%)	< 0.001
Lesion location					
Left main stem	0 (0.0%)	3 (1.7%)	33 (17.1%)	36 (6.6%)	< 0.001
Left anterior artery	95 (53.7%)	162 (91.5%)	185 (95.9%)	442 (80.8%)	< 0.001
Circumflex artery	53 (29.9%)	81 (45.8%)	150 (77.7%)	284 (51.9%)	< 0.001
Right artery	81 (45.8%)	107 (60.5%)	157 (81.3%)	345 (63.1%)	< 0.001
Lesion characteristics					
Bifurcated lesion	31 (17.5%)	99 (55.9%)	150 (77.7%)	280 (51.2%)	< 0.001
Trifurcated lesion	0 (0.0%)	4 (2.3%)	17 (8.8%)	21 (3.8%)	< 0.001
Ostial lesion	8 (4.5%)	6 (3.4%)	10 (5.2%)	24 (4.4%)	0.669
Lesion > 20 mm	11 (6.2%)	48 (27.1%)	108 (56.0%)	167 (30.5%)	< 0.0001
Lesion with thrombus	1 (0.6%)	1 (0.6%)	7 (3.6%)	9 (1.6%)	< 0.0001
Calcified lesion	3 (1.7%)	19 (10.7%)	53 (27.5%)	75 (13.7%)	< 0.0001
Total occlusion	1 (0.6%)	14 (7.9%)	64 (33.2%)	79 (14.4%)	< 0.0001
Chronic total occlusion	0 (0.0%)	3 (1.7%)	34 (17.6%)	37 (6.8%)	< 0.0001
Treated coronary					
3-Vessel intervention	1 (0.6%)	14 (7.9%)	39 (20.2%)	54 (9.9%)	< 0.0001
Left main stem	0 (0.0%)	2 (1.1%)	0 (0.0%)	2 (0.4%)	< 0.0001
Left anterior descending	84 (47.5%)	125 (70.6%)	91 (47.2%)	300 (54.8%)	< 0.0001
Circumflex	41 (23.2%)	34 (19.2%)	43 (22.3%)	118 (21.6%)	0.636
Right	68 (38.4%)	70 (39.5%)	84 (43.5%)	222 (40.6%)	0.573
Procedural characteristics					
Number of implanted stents	1.6 ± 0.9	2.1 ± 1.0	3.0 ± 1.3	2.2 ± 1.5	< 0.0001
Total stent length/patient (mm)	29.3 ± 16.8	41.1 ± 28.0	64.3 ± 30.7	66.3 ± 32.5	< 0.0001
Maximal pressure of stent deployment	13.8 ± 2.6	13.0 ± 2.8	14.8 ± 2.7	13.8 ± 1.7	< 0.0001
Patients with complete revascularization	147 (83.1%)	91 (51.4%)	25 (13%)	263 (48.1%)	< 0.0001
Use of glycoprotein IIb/IIIa inhibitors	0 (0.0%)	5 (2.8%)	16 (8.3%)	21 (4.8%)	< 0.0001
Post-procedural hospital stay, days	10.0 ± 5.9	9.6 ± 5.0	12.4 ± 9.6	10.6 ± 4.6	0.001
Intravascular imaging	0 (0.0%)	1 (0.2%)	7 (1.3%)	8 (1.5%)	< 0.0001

Value are n (%), mean ± SD

*SS* SYNTAX score

Only 36 (6.6%) patients had lesions involving the left main stem. 345 (63.1%) patients had right coronary artery DS > 50%. 441 (80.8%) patients had lesions located within the left anterior descending artery.

### Angiographic and procedural characteristics

Baseline lesion and procedural characteristics were shown in Table 2. Markers which increase lesion complexity, such as the presence of bifurcation lesions, long lesions, calcification, and total occlusions were all significantly higher in the SS_HIGH_ tertile.

In SS_HIGH_ tertile, the mean number of significant lesions for each patient was 3.3, received 3.0 stents with an average total stented length of 64.3 mm with the mean maximal air pressure 14.8 for stent deployment; all of which were significantly higher. Moreover, post-procedure in hospital stay was also significantly longer in the SS_HIGH_ tertile.

### SYNTAX score versus clinical SYNTAX score

Clinical outcomes across SS tertiles were shown in Table 3, the rate of MACCE, Target lesion failure (TLF, including cardiac death, target-vessel driven myocardial infarction, ischemia-driven target lesion revascularization), cardiac death, MI and any repeat revascularization were significant higher in the SS_HIGH_ tertile. During the follow-up period, 17 patients died. The rates of all-cause death in three group were 1.7% in SS_LOW_ tertile, 3.4% in SS_MED_ tertile and 4.1% in SS_HIGH_ tertile. Three patients died of definite non-cardiogenic cause (one died of traffic accident, one died of pulmonary carcinoma and another died of cerebral hemorrhage) were categorized into SS_LOW_ tertile.

**Table 3:** Clinical outcomes 2 years since date of procedural according to SS

Variable	SSlow ≤ 8 (177)	8 < SSmid < 18 (177)	18 ≤ SShigh (193)	*p* value
MACCE**	0 (0%)	12 (6.8%)	26 (13.5%)	< 0.0001
All-cause death	3 (1.7%)	6 (3.4%)	8 (4.1%)	0.385
Target vessel failure^#^	0 (0%)	4 (2.3%)	11 (5.7%)	0.003
Any repeat revascularization	0 (0%)	7 (4.0%)	14 (7.3%)	0.001
Stroke	0 (0%)	3 (1.7%)	0 (0%)	0.045
ARC stent thrombosis*	0 (0%)	1 (0.6%)	4 (2.1%)	0.094
Hospitalization due to angina pectoris	17 (9.6%)	17 (9.6%)	30 (15.6%)	0.113
Cardiac death	0 (0%)	3 (1.7%)	8 (4.2%)	0.016

Values are n (%), mean ± SD

*ACS* acute coronary syndrome

*Defined according to the Academic Research Consortium (ARC)

^#^Target lesion failure: cardiac death, target-vessel driven myocardium infraction, ischemia-driven target lesion revascularization (TLR)

**MACCE: cardiac death, any MI, any repeat revascularization and stroke

Clinical outcomes stratified CSS tertile were shown in Table 4. Comparing with those stratified by SS, CSS yielded similar results when comparing between the high and low risk group. However, in contrast to the SS analysis, there was significant difference in event rates for all-cause death between high and low CSS tertile.

**Table 4:** Clinical outcomes 2 years since date of procedural according to CSS

Variable	CSS_LOW_ (180)	CSS_MID_ (185)	CSS_HIGH_ (182)	*p* value
MACCE**	3 (1.7%)	7 (3.8%)	28 (15.4%)	< 0.0001
All-cause death	1 (0.6%)	4 (2.2%)	12 (6.6%)	0.003
Target vessel failure^#^	0 (0%)	4 (2.2%)	11 (6.0%)	0.002
Any repeat revascularization	3 (1.7%)	4 (2.2%)	14 (7.7%)	0.004
Stroke	0 (0%)	1 (0.5%)	2 (1.1%)	0.367
ARC stent thrombosis*	0 (0%)	4 (2.2%)	1 (0.5%)	0.078
Hospitalization due to angina pectoris	19 (10.6%)	25 (13.8%)	64 (11.7%)	0.563
Cardiac death	0 (0%)	1 (0.5%)	10 (5.5%)	< 0.0001

Values are n (%), mean ± SD

^*^Defined according to the Academic Research Consortium (ARC)

^#^Target lesion failure: cardiac death, target-vessel driven myocardium infraction, ischemia-driven target lesion revascularization (TLR)

^**^MACCE: cardiac death, any MI any repeat revascularization and stroke

### Kaplan–Meier analysis

**Fig. 2:**
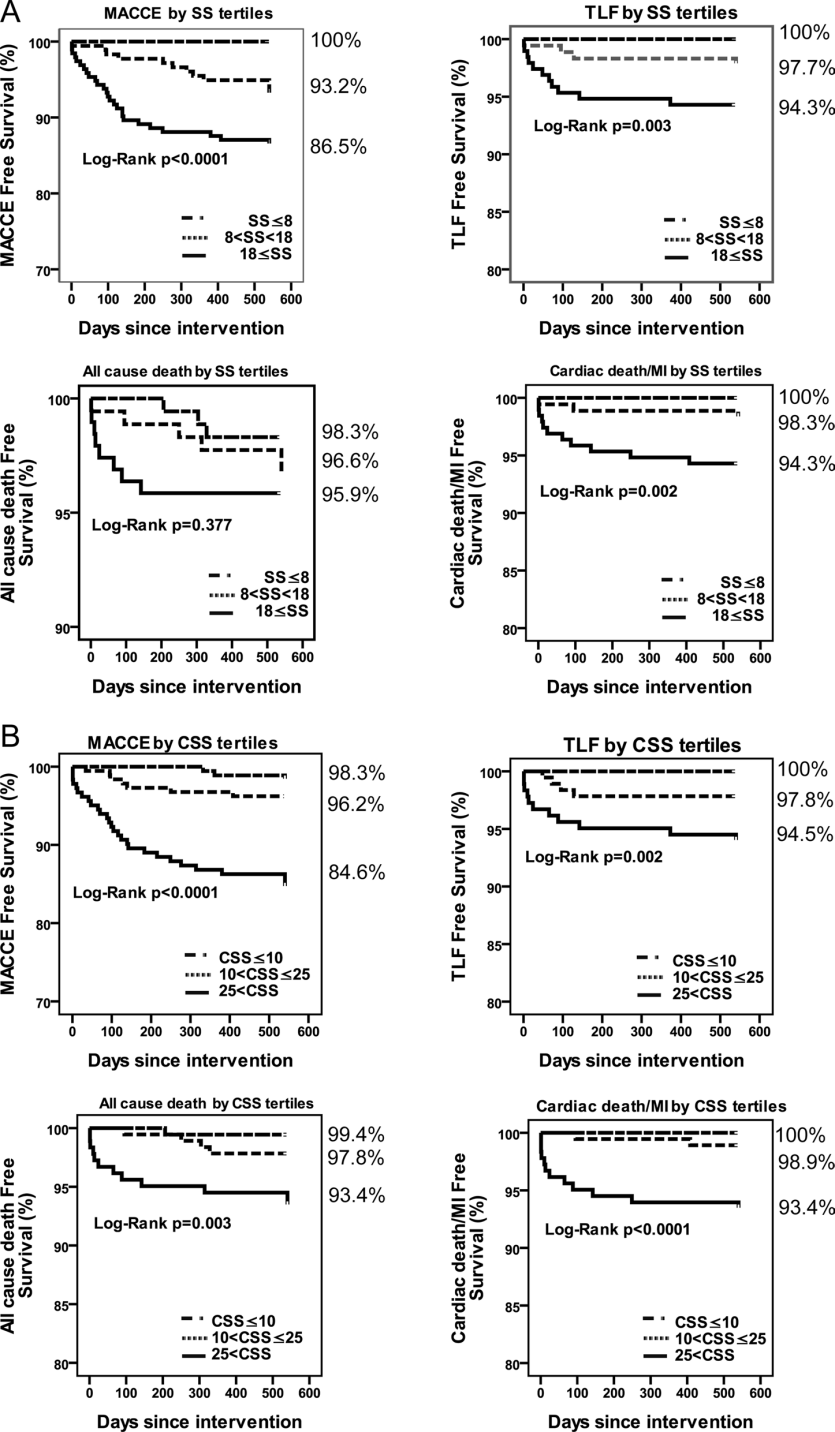
Clinical outcomes at 2-year follow up stratified by SS (**A**) and CSS (**B**) tertile. Kaplan–Meier curves are presented for major adverse cardiac and cerebral events, target lesion failure, all-cause death and cardiac death/myocardial infarction

As shown in Fig. 2A, the MACCE-free survival was significantly lower in SS_HIGH_ compared with SS_LOW_ (86.5% vs. 100%, *p* < 0.0001), such trend was also observed for survival rates of endpoints TLF (94.3% vs. 100%, *p* = 0.03) and cardiac death/MI (94.3% vs. 100%, *p* = 0.02). However, no trends were observed among the free survival rate of all-cause death (98.3% in the low, 96.6% in the median, 95.9% in the high, *p* = 0.377).

Clinical outcomes across by CSS tertile were shown in Fig. 2B. Survival curve of all-cause death was significantly higher in CSS_HIGH_ compare with CSS_LOW_ (Fig. 2B). The survival rate of MACCE, TLF and cardiac death/MI were also significantly higher in CSS_HIGH_ compared with CSS_LOW_ tertile, similar to results from SS tertile.

### The ROC curve analysis

**Fig. 3:**
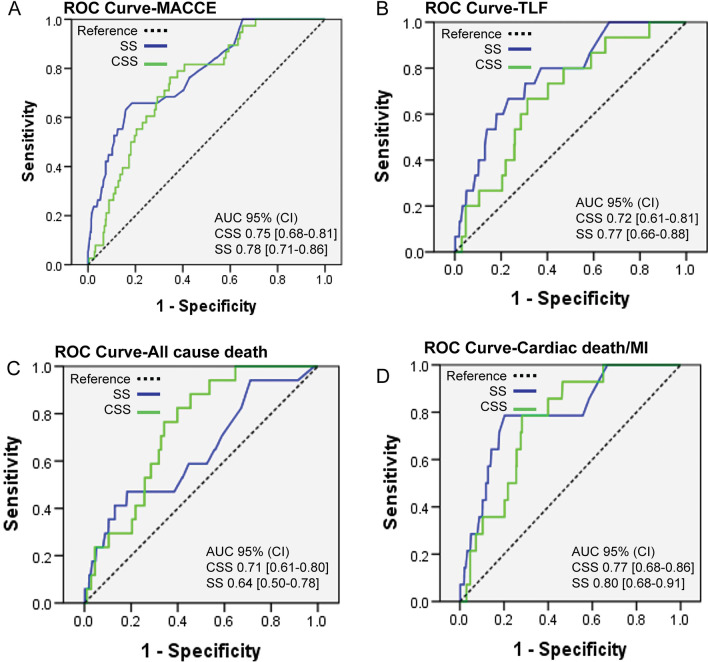
Receiver operating characteristic (ROC) curves for SS and CSS. (**A)** major adverse cardiac and cerebral events (**B)** target lesion failure (**C**) all-cause death (**D**) cardiac death/myocardium infarction. *AUC* area-under-the-curve, *CI* confidence interval, *SS* SYNTAX score, *CSS* clinical SYNTAX score

The receiver operating characteristic (ROC) curves for MACCE, TLF, all-cause death and cardiac death/MI during 2-year follow-up were shown in Fig. 3A–D. In this analysis, the areas under the curves (AUC) of both scores were significantly (both *p* < 0.001) higher than the area of diagnostic indifference. However, we found the AUC is not larger for CSS MACCE (AUC SS, 0.78; AUC CSS, 0.75), TLF (AUC SS, 0.77; AUC CSS, 0.72) and cardiac death/MI (AUC SS, 0.80; AUC CSS, 0.70). The AUC for all-cause death by CSS was slightly larger comparing to SS but not significant (AUC SS, 0.64; AUC CSS, 0.71; *p* = 0.23) **(**Fig. 3C). The optimal cut-points were selected using the common method of Youden index method. Optimal cut-points were calculated as 21.00 for SS and 45.35 for CSS. We calculated the Youden index, positive and negative prediction values. Youden index is 0.38 and 0.46 for SS and CSS, respectively. Positive/negative prediction value is 20.62%/96.00% for SS and 23.47%/96.65% for CSS. These results suggested comparable prediction ability comparing SS and CSS in our study population.

### Cox multivariable analysis

The results of COX multivariable analysis were shown in Table 5. After adjust confounding factors including age, sex, DM, presentation with ACS, number stent implanted, the SS remained as an independent predictor for MACCE [1.101 (1.070–1.134), *p* < 0.0001]. The CSS remained as an independent predictor for all-cause death at 2-year follow up [1.012 (1.000–1.024), *p* < 0.05].

**Table 5:** COX multivariable analysis

Clinical outcome	Hazard ratio for CSS (95% CI)	*p* value	Hazard ratio for SS (95% CI)*	*p* value
All-cause death	1.012 (1.000–1.024)	0.049	1.049 (1.005–1.095)	0.027
Target lesion failure	1.014 (1.001–1.028)	0.034	1.090 (1.042–1.140)	< 0.0001
Cardiac death/MI	1.018 (1.004–1.030)	0.006	1.100 (1.049–1.153)	< 0.0001
MACCE	1.017 (1.009–1.022)	< 0.0001	1.101 (1.070–1.134)	< 0.0001
Any MI	1.024 (1.002–1.047)	0.034	1.105 (1.010–1.209)	0.03
Any repeat revascularization	1.018 (1.008–1.029)	< 0.0001	1.107 (1.064–1.152)	< 0.0001

*CI* confidence interval, *HR* hazard ratio, *ACS* acute coronary syndrome, *MACCE* major adverse cardiac and cerebral events, *SS* SYNTAX score, *CSS* clinical SYNTAX score

### Reclassifying from SS into CSS tertile

**Fig. 4:**
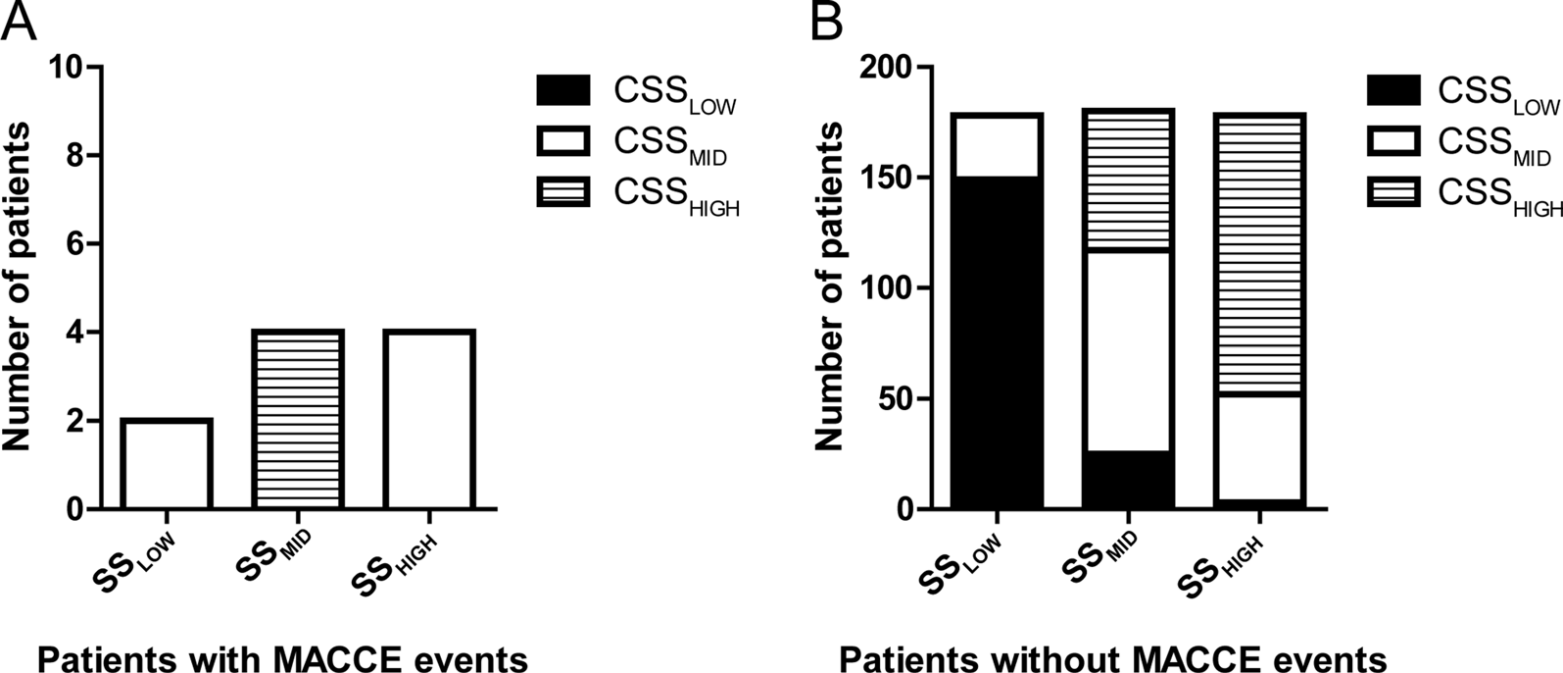
Reclassification of patients from SS tertiles to CSS tertiles

When reclassifying patients with MACCE from SS tertiles into CSS tertiles, 4/10 (40.0%) patients with events were moved to higher risk categories (upward) and 6/10 (60.0%) to lower risk categories (downward) with a net difference of 20.0%. In patients without MACCE, 92/537 (17.1%) were moved downward and 77/537 (14.3%) upward, with a net difference of 2.8%, as shown in Table 6 and Fig. 4.

**Table 6:** MACCE reclassification into CSS tertile

	CSS_LOW_	CSS_MID_	CSS_HIGH_	Total
Patients with MACCE events				
SS_LOW_	0	0	0	0
SS_MID_	2	0	4	6
SS_HIGH_	0	4	0	4
Total	2	4	4	10
Patients without MACCE events				
SS_LOW_	149	25	3	178
SS_MID_	29	92	49	160
SS_HIGH_	0	63	126	189
Total	178	181	178	537

## Discussion

The prognostic value of the SS have been well proved both in large-scale registry or a randomized controlled trial [6]. The main findings of this study indicated that both SS and CSS are valuable in the risk stratification of long-term clinical outcomes in an all-comers Chinese population receiving drug-eluting stents. Both scores were identified as independent predictors for MACCE but the CSS was also an independent predictor for all cause death. Although the SS was an angiographic scoring system that was developed in an attempt to risk stratification of patients undergoing revascularization of the left main coronary artery and/or the 3 main coronary arteries [17]. However, we found that other than patients involved LM undergoing PCI, the SS still remained as an independent predictor for MACCE in low and moderate risk patients. Therefore, the SS also has the ability to predict the clinical outcomes in low and median risk patients with PCI.

Consistent with previous results, this study also showed that the SS have the ability to stratify patients into different risk categories of adverse events after PCI [15]. However, we showed that the event rate of MACCE was the highest in SS_HIGH_ tertile, but the event rate of all-cause death was not statistically different. In addition, some studies argue that the SS only plays a partial role in predicting long-term adverse events after PCI and calls for incorporation of traditional well-recognized clinical risk factors in risk evaluation [6]. To address this question, several scoring systems have been developed for patients underwent PCI, however, few are being used by physicians in clinical practice. These scoring systems include the AHA/ACC score (American College of Cardiology/American Heart Association) [18], EuroSCORE (European System for Cardiac Operative Risk Evaluation) [19], global risk classification [20] and Parsonnet score [21], et al. These risk models used a selection of clinical variables that had been identified as predictors of adverse outcome in patients with PCI.

Recently, Garg et al. combined the SS with modified ACEF score (incorporating age, left ventricular ejection fraction, and creatinine clearance) to produce CSS. They found that, the AUC for CSS was significantly larger compared to that of SS regarding all-cause death (0.66 vs. 0.58, *p* < 0.001) [22] suggesting better accuracy. After the study of Garg et al., there have been studies comparing the prognostic value of the SS and CSS on the outcomes of ACS patients undergoing PCI [15, 23]. In the study of He et al*.*, CSS demonstrated significantly improved capability in predicting 2-year cardiac death as analyzed by receiver-operating characteristic curve, but not for prediction of MACE [23]. Girasis et al. reported AUC of CSS was significantly larger compared to that of SS regarding cardiac death and all-cause mortality, but not significant for MACE [15]. In our study, SS showed a tendency of larger AUC for CSS in terms of all cause death, but not for MACCE, TLF and cardiac death/MI. This is mostly likely because, in this study, only a small percentile of patients had lesions involving the left main (LM) stem, but more than half of patients had 1-vessel or 2-vessel disease, and a majority of the enrolled population are at low or moderate risk comparing to other studies whether most patients are at higher risk.

## Study limitations

The study has the following limitations. First, this study was single center design, intra- and inter-observer variability, which was inherent to coronary angiography cannot be ruled out. Secondly, the enrollment number was not big enough. Thirdly, the functional information of coronary circulation [24], like the fractional flow reserve (FFR) which was known to impact the clinical outcome, is lacking [25]. Finally, the retrospective design of the study, where patients have been categorized to be more clinically suitable for PCI instead of CABG may also pose an important bias.

## Conclusion

Both SS and CSS containing clinical information were able to stratify risk of clinical outcomes in all-comers Chinese population treated with PCI using drug-eluting stents. In this study of Chinese patients underwent PCI, CSS performed comparable ability in predicting outcomes comparing with the SS.

## Data Availability

All original data used to support the findings of this study are available from the corresponding author upon reasonable request.
